# The Prevalence of and Factors Associated with Disordered Eating Among Adult Athletes in Italy and Lebanon

**DOI:** 10.3390/nu17010191

**Published:** 2025-01-05

**Authors:** Valentina Cavedon, Dima Kreidieh, Chiara Milanese, Leila Itani, Massimo Pellegrini, Dana Saadeddine, Elisa Berri, Marwan El Ghoch

**Affiliations:** 1 Department of Neurosciences, Biomedicine and Movement Sciences, University of Verona, 37134 Verona, Italy; valentina.cavedon@univr.it (V.C.); chiara.milanese@univr.it (C.M.); 2 Department of Nutrition and Dietetics, Faculty of Health Sciences, Beirut Arab University, P.O. Box 11-5020 Riad El Solh, Beirut 11072809, Lebanon; d.kraydeyeh@bau.edu.lb (D.K.); l.itani@bau.edu.lb (L.I.); 3 Center for the Study of Metabolism, Body Composition and Lifestyle, Department of Biomedical, Metabolic and Neural Sciences, University of Modena and Reggio Emilia, 41125 Modena, Italy; massimo.pellegrini@unimore.it (M.P.); dana.saadeddine@unimore.it (D.S.); 4 Degree Course of Dietetics, Innovation and Research Training Service, Azienda Ospedaliero-Universitaria di Modena, 41124 Modena, Italy; elisa.berri@unimore.it; 5 Department of Primary Care, Azienda Unità Sanitaria Locale—IRCCS di Reggio Emilia, 42123 Reggio Emilia, Italy

**Keywords:** dieting, eating disorders, sport competitions, overtraining, physical exercise, physical fitness and performance

## Abstract

Background/Objectives: Disordered eating (DE) is a wide-spectrum condition, represented by altered eating patterns, behaviors, and attitudes aimed at controlling food intake, body weight, and shape, which does not necessarily satisfy the diagnostic criteria for an eating disorder of clinical severity. DE is frequently reported among athletes, but its prevalence and associated factors have not been fully elucidated. In this study, we intended to assess the prevalence of DE among adult athletes from different sports disciplines in Italy and Lebanon and to identify the factors associated with DE. Methods. A validated questionnaire (Eating Attitude Test [EAT-26]) was administered to determine the prevalence of DE, which was indicated by a score ≥ 17. Sport-related information, such as the type of sport, level of competition, training volume, and years of athletic experience, was also collected. Results: Among the total sample of 881 athletes, 78 were identified as having DE, with a prevalence of 6.1% (7.8% of females and 4.9% of males) in Italian athletes and 21.3% (27.3% of females and 17.0% of males) in Lebanese athletes. In addition, among male athletes, the risk of having DE was more than threefold in those practicing weightlifting or bodybuilding (odds ratio [OR] = 3.23; 95% confidence interval [CI] = 1.03–10.08, and *p* < 0.05), while females with more athletic experience had almost 10% less risk of having DE (OR = 0.92; 95%CI = 0.86–0.98, and *p* < 0.05). Conclusions: DE is a prevalent condition among athletes. Therefore, it is crucial that sports federations and committees consider adopting standardized practical guidelines that focus on routinely screening for the early identification of DE in this population and implementing strategies for its timely management. In the future, longitudinal studies are also needed to clarify the impact of DE on athletes’ clinical condition as well as their physical fitness and sports performance.

## 1. Introduction

Athletic performance refers to the accomplishment of an athlete in a specific sports discipline during training and/or official competitions [1]. Improving their performance to excel in their respective sport(s) is the main goal of sportsmen and sportswomen, as well as their coaches and teams [1]. For this reason, the identification of factors or determinants associated with or related to better or poorer athletic performance is vital in any sports discipline and remains a priority for any athlete [2,3,4,5]. For instance, an eating disorder is a clinical condition characterized by its central and specific psychopathology, namely, the over-evaluation of eating, body shape, and weight and their control through dysfunctional behaviors (i.e., dieting, self-induced vomiting, binging, excessive physical exercising, body checking, and avoidance) [6], and may adversely impact the individual’s physical health [7] as well as psychosocial wellbeing [8,9]. These complications appear to arise through different mechanisms, such as malnutrition and low body weight [10,11], as well as dysfunctional purging behaviors [12], which often operate concurrently and interact with each other and with the eating disorder psychopathology.

In this context, at the general population level, the lifetime prevalence of eating disorders ranges between 1% and 3% (i.e., 1.89%) and affects more females (i.e., 2.58%) than males [13], especially during adolescence and young adulthood [14]. Along this line, it is well-documented that eating disorders are more prevalent among athletes in comparison to individuals in the general population [15] as large-sample and well-designed studies on athletes found an overall prevalence of eating disorders of 13.5%, which is far higher than that reported for the general population [16]. Moreover, it is also known that eating disorders in athletes not only affect their health outcomes but appear to harm their physical fitness and sports performance as well [17,18].

In relation to the above, disordered eating (DE) is a subclinical condition that encompasses a broad range of unhealthy eating attitudes and behaviors that do not necessarily satisfy the diagnostic criteria for an eating disorder of clinical severity [19]. However, it certainly represents a risk factor for eating disorders in terms of its onset and development [20], which can contribute to psychological and physical stress, and also seems to affect the sports performance of an athlete [21]. Moreover, when these are added to cultural factors (i.e., beliefs and attitudes), this may lead to differences in the prevalence of DE across different countries [22]. However, the effects of these factors are usually ignored and underestimated. Therefore, the early screening and identification of DE and the related factors/determinants are important as a prevention strategy for promoting the health of athletes in order to protect them from developing an eating disorder, as well as to preserve their athletic and sports performance. However, despite the fact that several investigations have been conducted on DE in athletes, little is still known about its prevalence, especially in certain sports disciplines as well as in various geographical areas and regions (i.e., countries) due to a paucity of studies [23].

Accordingly, this study first aims to screen for the prevalence of DE in a sample of competitive athletes composed of both males and females from two nations, Italy and Lebanon. Second, it seeks to detect any potential differences in its prevalence between sexes and countries since one of the two countries is Western and the other is Middle Eastern. Finally, it attempts to identify the sport-related factors that are more likely to be associated with DE.

## 2. Materials and Methods

### 2.1. Study Design and Population

**Figure 1:**
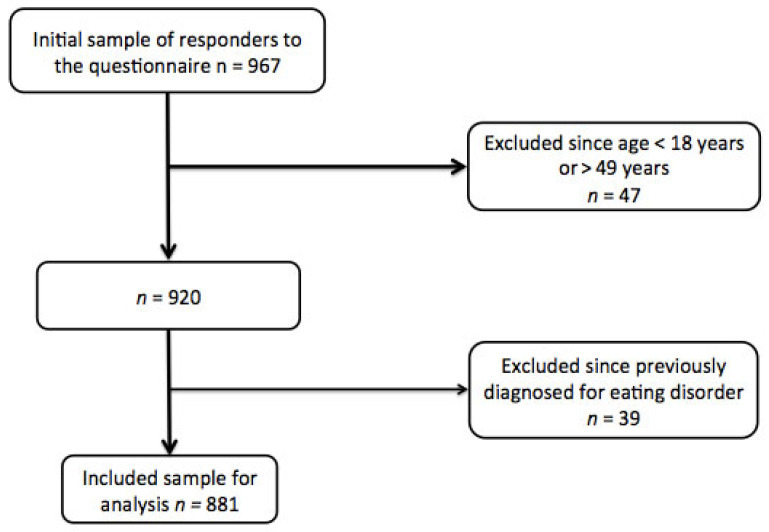
Flowchart and shift work analysis.

This cross-sectional study was conducted between May and October 2024. We initially enrolled 968 athletes of both sexes at different competitive levels and sports disciplines from several national sports associations and academies in Italy and Lebanon. The inclusion criteria were as follows: (i) being an adult (<50 years) male or female and (ii) practicing a sport discipline at a competitive level and identifying as a competitive athlete (i.e., at the level of a city/province or region/district or at a national or international level). The exclusion criteria were (i) an age < 18 years or >50 years, (ii) practicing a sport as a hobby and not at a competitive level (hence not being considered an athlete), (iii) athletes who were unable or unwilling to complete the questionnaires entirely, and (iv) athletes who had received a diagnosis for an eating disorder according to the Diagnostic and Statistical Manual of Mental Disorders, Fifth Edition (DSM-5), or were currently under specialized treatment for any form of eating disorder (Figure 1). This investigation was approved by the Institutional Review Board (IRB) of Beirut Arab University (BAU) with the number 2024-H-0190-HS-M-0619, and all participants provided informed written consent.

### 2.2. Questionnaires

#### 2.2.1. General Questionnaire

This questionnaire was administered to participants in order to retrieve general information regarding their medical history and demographic and social conditions, such as age, sex, weight, height, educational level, medical history, and current health status related to eating disorders, and whether the athlete had received a diagnosis or underwent or was currently under treatment for eating disorders. Questions were also asked about information related to sport, such as:

- Type of sport: according to the frequencies in our sample, the athletes were classified according to their sports discipline as competing in either team sports (i.e., mainly ball games such as football, basketball, volleyball, handball, etc.) or individual ones, categorized as athletics, aesthetics, weightlifting/bodybuilding, and others.
- Level of competition: according to the level of competition at which they practice their sport, the athletes were classified at four different levels: city/province, region/district, national, or international.
- Years of athletic experience: the duration for which the athlete has been practicing a specific sport at a competitive level, expressed in years.
- Training volume: the number of hours per week spent in training sessions.

#### 2.2.2. Eating Attitudes

The athletes’ eating attitudes were assessed using the Eating Attitudes Test (EAT-26) in order to identify DE [24]. The EAT-26 is a self-reported questionnaire widely utilized on an international scale and is a highly accepted tool for screening for DE in several groups from the general population (i.e., high school and college) as well as athletes [25,26,27,28,29,30,31,32,33,34,35,36]. The questionnaire includes 26 questions, with six response options: always, very often, often, sometimes, seldom, and never. In this study, a score of 17 or higher was considered the threshold for indicating DE, as recently suggested based on non-clinical samples [37]. The EAT-26 questionnaire has been culturally validated and widely used across the Italian and Lebanese populations [38,39].

The general and EAT-26 questionnaires were administered through a Google Forms link, and data were collected in accordance with the CHERRIES criteria for web-based surveys. In the Lebanese population, the original English version of the EAT-26 questionnaire [24] or its Arabic validated version [38] was used according to the participant’s preference. Among the Italian athletes, the Italian validated version of EAT-26 was distributed [39]. After the identification of the athletes through their sports association, they were informed about the protocol procedures and asked about their willingness to participate in this study. Those eligible were asked to give their consent to participate, and the link was forwarded to them through their association.

### 2.3. Statistical Analysis

Descriptive statistics are presented as means and standard deviations for continuous variables and as frequencies and proportions for categorical variables. Student’s t-test was used for mean comparison, and the chi-squared test for independence was utilized for categorical variables. Pearson’s correlation coefficient and scatter plots were used to illustrate the association between independent variables and the EAT-26 score where necessary. Prevalence rates and distributions are presented as bar graphs. Multivariate logistic regression analysis was employed to assess the determinants of DE while adjusting for potential confounders. The significance for all tests was considered at *p* < 0.05. All statistical analyses were conducted with Statistical Package for the Social Sciences (SPSS) software version 26 (2019) [40].

## 3. Results

A sample of 881 adult athletes aged between 18 and 49 years was included in this study. The sample consisted of 520 males (59.0%) and 361 females (41.0%). The mean age was 24.8 ± 6.5 years, and most of them were unmarried (84.4%). Of the entire sample, 12.4% reported competing on an international level, 33.6% on a national level, 27% on a regional level, and 27% on a city level. The mean training volume was 9.3 ± 6.8 h/week. There were no significant differences between males and females related to age, marital status, competitive level, or training volume (*p* > 0.05). The majority of participants were Italian (81.8%), while only 18.2% were Lebanese (Table 1).

**Figure 2:**
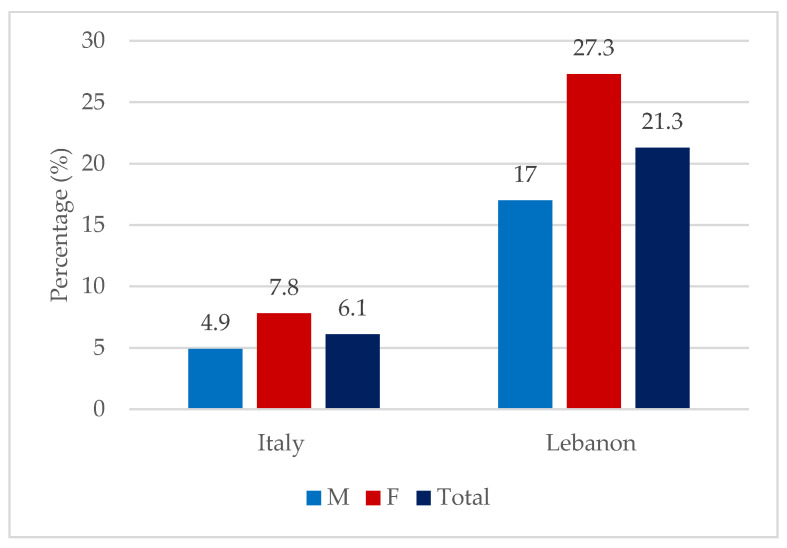
Prevalence of DE among males and females stratified by country of origin.

Among the entire sample, a total of 78 individuals were identified as having DE, as assessed by EAT-26 with a score ≥ 17 [37]. In total, 41 were females (52.6%) and 37 were males (47.4%). The overall prevalence of DE was higher among Lebanese athletes, affecting 21.3% compared to 6.1% among Italian athletes. Accordingly, the prevalence among Lebanese female (27.3%) athletes was also higher compared to Italian female (7.8%) athletes; similarly, DE prevalence was higher among Lebanese male (17.0%) athletes compared to Italian male athletes (4.9%) (Figure 2).

**Figure 3:**
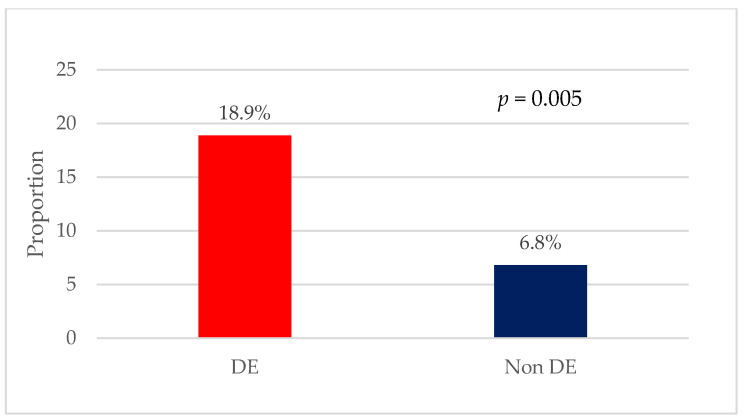
Proportion of weightlifting and bodybuilding among male athletes with or without DE.

**Figure 4:**
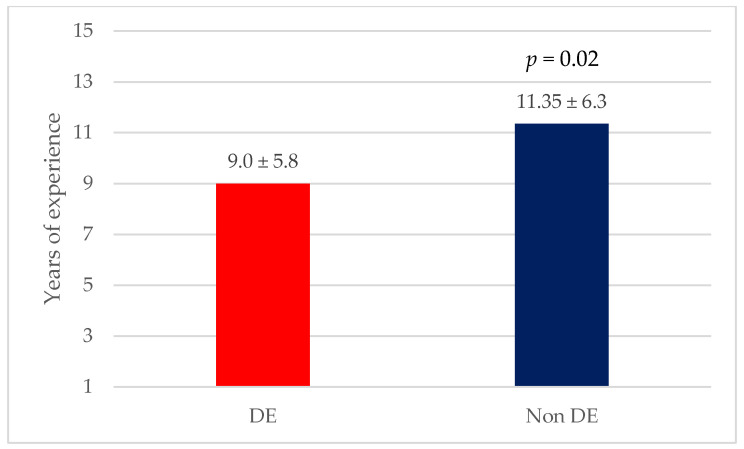
Mean years of experience among female athletes with or without DE.

Table 2 presents the characteristics of the study participants by EAT-26 score category. Male athletes with DE were older than athletes without DE (28.8 ± 8.9 years vs. 24.7 ± 6.1 years, *p* = 0.009) and had a higher body mass index (BMI) (24.8± 4.1 kg/m^2^ vs. 23.4 ±2.7 kg/m^2^, *p* = 0.048). They had a higher level of education (62.2% vs. 37.1%, *p* = 0.003) and were more likely to practice weightlifting and bodybuilding (18.9% vs. 6.8%, *p* = 0.005) (Figure 3) or aesthetic sports (10.8% vs. 3.1%, *p* = 0.005) compared to athletes without DE. Female athletes with DE did not differ in terms of age from those without DE. However, they had a higher BMI (22.8± 2.9 kg/m^2^ vs. 21.6± 2.9 kg/m^2^, *p* = 0.019) compared to those without DE, as well as fewer years of athletic experience (9.0 ± 5.8 years vs. 11.4 ± 6.3 years, *p* = 0.02) (Figure 4).

After adjusting for confounders, logistic regression analysis confirmed the above findings, where male athletes were almost three times more likely to have DE if they were practicing weightlifting and bodybuilding (odds ratio [OR]= 3.23; 95% confidence interval [CI] = 1.03–10.08, and *p* < 0.05), while female athletes with more athletic experience were 8% less likely to have DE compared to those with less athletic experience (OR = 0.92; 95% CI = 0.86–0.98, and *p* < 0.05) (Table 3).

## 4. Discussion

The present study aimed to provide benchmark data on the occurrence of DE among athletes, and it revealed three main findings.

### 4.1. Main Findings and Concordance with Previous Literature

First, on a general scale across the entire sample, our investigation reported a relatively high prevalence of DE, which was estimated to be approximately 10% regardless of sex and country of origin. When stratified by country, DE prevalence was almost 6.0% among Italian athletes and 21.0% among Lebanese athletes. Within each country, DE was more prevalent among females (7.8% and 27.3%) compared to males (4.9% and 17.0%) in the Italian and Lebanese samples, respectively. The prevalence of DE reported in our study among Italians was similar to that for Greek athletes (5.1%) [41] but lower than that for German athletes (16%) [42]. On the other hand, the prevalence in Lebanese athletes was similar to that in athletes from Finland (18%) [43], Turkey (19%) [44], and Australia (23%) [45] but significantly lower than that reported for athletes in France (33%) [46], Jordan (34%) [47], and Saudi Arabia (36%) [48].

Second, another aspect of particular interest is the higher prevalence of DE among Lebanese athletes by almost four times when compared to Italian ones (i.e., the Middle Eastern vs. Western population). Our finding is in line with those published in a recent systematic review, meta-analysis, and meta-regression, which reported that Western countries had a significantly lower prevalence of DE among athletes than Eastern countries, but the authors were not able to explain this difference [49]. Although we are also not in a position to identify the reason behind this discrepancy in prevalence between Lebanon, considered a Middle Eastern country, and Italy, which is a Western one, we may speculate that this can be attributed to at least two reasons. First, athletes in Italy mandatorily receive a rigorous medical evaluation to determine their eligibility to practice sports at a competitive level, and without this certification, the athlete cannot participate in either training or competitions. This medical evaluation does not specifically screen for eating disorders or DE, but it can indicate the existence of any related medical complication and will certainly exclude these individuals from the possibility of practicing sport on a competitive level. Another reason may be that studies conducted in Eastern countries usually have small sample sizes and may not be representative of all athletes in these countries as the sample may be overconcentrated with individuals with DE. Its prevalence in these populations may therefore be overestimated. However, we cannot exclude the existence of other factors that we did not investigate in our study, such as culture, which has been identified as a significant contributing factor in the development and evolution of eating disorders and DE [22]. This may explain the differences in their prevalence across countries of different cultures [50].

Third, we were able to identify two sport-related factors associated with a higher/lower risk of DE. Namely, female athletes with more athletic experience, expressed in years, were less likely to have DE. In terms of sports discipline, practicing bodybuilding or weightlifting was a risk factor for DE among males. These two sports usually require more pronounced muscularity, and this can fuel the risk of having an eating disorder (i.e., bigorexia) in this population (i.e., males), especially if the central psychopathology is more focused on muscle dysmorphia (i.e., feeling too small or not muscular enough despite having a normal build) [51].

### 4.2. Clinical Implications

These findings have certain implications in the sporting environment. First, awareness should be raised regarding the high prevalence of DE among athletes, who should be encouraged to openly refer to and discuss this condition with their management (i.e., coach, trainer, etc.) as well as with the health professionals related to sports (i.e., sports doctors, dietitians, psychologists, specialists in motor sciences, physiotherapists, etc.), who should be all well-educated and trained on the topic in order to reduce/avoid the stigma towards these athletes. Second, with this aim in mind, sports committees and federations should consider adopting practical guidelines [52] that include screening tools for DE as a routine and regular procedure in all sports and at all competitive levels, which could contribute to its early identification, as well as establishing predetermined preventive and therapeutic pathways within action programs to promote the management of DE to avert its evolution into an eating disorder of clinical severity.

### 4.3. Strengths and Limitations

This study has certain strengths. Most importantly, it is one of the very few studies that has assessed DE among athletes in Italy as well as in Lebanon. In fact, as far as we are aware, only one investigation has been conducted in Italy, on nearly 200 athletes practicing only ballet, gymnastics, and bodybuilding [53], and so far, no research has been performed in Lebanon on this topic. Second, the use of the EAT-26 questionnaire to assess DE can also be considered an advantage since it has been extensively used among athletes [42,54,55]. Third, the inclusion of a large sample of athletes competing at high levels, derived from two culturally different countries (a Middle Eastern one and a Western one), is also a strength.

However, this paper does have certain limitations. First and foremost, not all types of sports were represented (i.e., cricket, baseball, team sports, etc.) in our relatively small sample size; therefore, our findings lack external validity and cannot be generalized and extended to all sports disciplines. Second, our study relies on voluntary participation by athletes, which may lead to selection bias. Third, we did not explore psychological and socio-cultural factors that may have explained the differences in the prevalence of DE between the two countries (i.e., Italy vs. Lebanon). Fourth, the cross-sectional design of this study should be considered another limitation since it does not allow for the detection of the potential negative impact of DE on health outcomes (i.e., medical and psychosocial) or whether it may adversely affect sports performance in these athletes or evolve into an eating disorder in the long term.

### 4.4. New Directions for Future Research

Future research on this topic is necessary and should be conducted along the following directions: First, studies with a similar aim and design to ours are needed in other countries and geographical regions where data on the prevalence of DE are lacking. Second, large sample investigations that include other sports disciplines that were not represented in our study are needed. Third, studies that enable the identification of new relevant factors that are associated with a higher/lower risk of DE, such as anthropometric and body composition variables, cardio-metabolic biomarkers, and socio-demographic as well as sport-related variables, are necessary in order to facilitate the early identification of DE, promote its management, and prevent its evolution into an eating disorder of clinical severity. Fourth, qualitative studies should be conducted to identify psychological and cultural factors that may influence perceptions of body image and eating habits in different cultures in order to help explain differences in the prevalence of DE across countries. Finally, longitudinal evaluations that clarify the impact of DE on athletes’ clinical condition (medical or psychological) as well as on the physical fitness and sports performance of an athlete in the short, intermediate, and long terms are also needed.

## 5. Conclusions

In conclusion, our findings provide evidence regarding the high prevalence of DE among athletes, regardless of their sex or ethnicity. For this reason, policies should be established in order to take action at different levels, such as education and awareness campaigns on the topic (i.e., eating disorders) to increase knowledge and reduce stigmatization, the implementation of effective screening systems to identify athletes at a higher risk, and finally, structuring specific programs for the early management of DE.

## Figures and Tables

**Table 1:** Characteristics of the study participants by sex ^¥^.

	Total (n = 881)	Males (n = 520)	Females (n = 361)	Significance
Age	24.8 (6.5)	25.1 (6.4)	24.4 (6.6)	*p* = 0.158
Education				X^2^ = 0.650; *p* = 0.420
Lower education	529 (60.0)	318 (61.2)	211 (58.3)	
Higher education	352 (40.0)	202 (38.8)	150 (41.6)	
Marital status				X^2^ = 2.366; *p* = 0.124
Not married	744 (84.4)	431 (82.9)	313 (86.7)	
Married/cohabiting	137 (15.6)	89 (17.1)	48 (13.3)	
Competitive level				X^2^ = 7.717; *p* = 0.052
City/provincial	238 (27.0)	152 (29.2)	86 (23.8)	
National	296 (33.6)	161 (31.0)	135 (37.4)	
Regional	238 (27.0)	149 (28.7)	89 (24.7)	
International	109 (12.4)	58 (11.2)	51 (14.1)	
Nationality				X^2^ = 0.006; *p* = 0.938
Italian	721 (81.8)	426 (81.9)	295 (81.7)	
Lebanese	160 (18.2)	94 (18.1)	66 (18.3)	
Years of athletic experience	11.5 (6.5)	11.7 (6.6)	11.1 (6.3)	*p* = 0.146
Volume of training (hours/week)	9.3 (6.8)	9.5 (7.4)	9.1 (5.9)	*p* = 0.425

^¥^ Values are means (SD) for continuous variables and frequencies (%) for categorical variables.

**Table 2:** Characteristics of study participants by categories of EAT-26 score ^¥^.

	Males			Females		
	EAT-26 < 17 (n = 483)	EAT-26 ≥ 17 (n = 37)	Significance	EAT-26 < 17 (n = 320)	EAT-26 ≥ 17 (n = 41)	Significance
Age (years)	24.7 (6.1)	28.8 (8.9)	*p* = 0.009	24.3 (6.5)	25.2 (7.2)	*p* = 0.448
BMI (kg/m^2^)	23.4 (2.69)	24.8 (4.1)	*p* = 0.048	21.6 (2.9)	22.8 (2.9)	*p* = 0.019
Level of education			X^2^ = 9.116; *p* = 0.003			X^2^ = 0.995; *p* = 0.318
Lower education	304 (62.9)	14 (37.8)		190 (59.4)	21 (51.2)	
Higher education	179 (37.1)	23 (62.2)		130 (40.6)	20 (48.8)	
Marital status			X^2^ = 0.091; *p* = 0.762			X^2^ = 0.072; *p* = 0.789
Not married	401 (83.0)	30 (81.1)		278 (86.9)	35 (85.4)	
Married or co-habiting	82 (17.0)	7 (18.9)		42 (13.1)	6 (14.6)	
Type of sport			X^2^ = 14.817; *p* = 0.005			X^2^ = 2.363; *p* = 0.669
Team sports	258 (53.4)	14 (37.8)		128 (40.0)	15 (36.6)	
Athletics	91 (18.8)	8 (21.6)		91 (28.4)	11 (26.8)	
Aesthetics	15 (3.1)	4 (10.8)		35 (10.9)	7 (17.1)	
Weightlifting or bodybuilding	33 (6.8)	7 (18.9)		14 (4.4))	3 (7.3)	
Other	86(17.8)	4 (10.8)		52 (16.3)	5 (12.2)	
Competitive level			X^2^ = 2.763; *p* = 0.430			X^2^ = 1.418; *p* = 0.701
City/province	142 (19.4)	10 (27.0)		79 (24.7)	7 (17.1)	
Regional	142 (29.4)	7 (18.9)		79 (24.7)	10 (24.4)	
National	146 (30.2)	15 (40.5)		117 (36.6)	18 (43.9)	
International	53 (11.0)	5 (13.5)		45 (14.1)	6 (14.6)	
Years of athletic experience	11.7 (6.5)	12.5 (8.7)	*p* = 0.592	11.4(6.3)	9.0 (5.8)	*p* = 0.020
Volume of training (hours/week)	9.3 (7.3)	11.6 (8.5)	*p* = 0.113	9.1 (6.0)	9.4 (4.7)	*p* = 0.697

^¥^ Values are means (SD) for continuous variables and frequencies (%) for categorical variables.

**Table 3:** Odds ratio and 95% confidence intervals for determinants of DE among male and female athletes (n = 881).

	Males	Females
	OR (95%CI)	
Age	1.06 (0.99–1.13)	1.01 (0.95–1.08)
BMI	1.09 (0.98–1.21)	1.13 (0.99–1.28)
Level of education		
Lower education	1.00	1.00
Higher education	2.23 (1.04–4.81) *	1.45 (0.66–3.18)
Type of sport		
Team sports	1.00	1.00
Athletics	1.12 (0.38–3.29)	0.70 (0.25–1.95)
Aesthetics	3.69 (0.98–13.93)	1.78 (0.62–5.07)
Weightlifting or bodybuilding	3.23 (1.03–10.08) *	0.78 (0.17–3.62)
Other	0.66 (0.20–2.23)	0.55 (0.17–1.76)
Competitive level		
City/province	1.00	1.00
Regional	0.76 (0.27–2.17)	1.98 (0.66–5.92)
National	1.39 (0.56–3.43)	2.05 (0.75–5.60)
International	0.75 (0.19–2.95)	2.62 (0.65–10.58)
Years of athletic experience	0.99 (0.94–1.05)	0.92 (0.86–0.98) *
Volume of training (hours/week)	1.03 (0.99–1.07)	1.01 (0.95–1.08)

* Significant at *p* < 0.05.

## Data Availability

The original contributions presented in this study are included in the article. Further inquiries can be directed to the corresponding author(s).
